# N-Stearoyl-L-Tyrosine Inhibits the Senescence of Neural Stem/Progenitor Cells Induced by A*β*_1–42_ via the CB2 Receptor

**DOI:** 10.1155/2016/7419389

**Published:** 2016-01-10

**Authors:** Wen-Qing Li, Ze-jian Wang, Sha Liu, Yue Hu, Ming Yin, Yang Lu

**Affiliations:** ^1^School of Pharmacy, Shanghai Jiao Tong University, Shanghai 200240, China; ^2^Shanghai Jiao Tong University School of Medicine, Shanghai 200025, China

## Abstract

Alzheimer's disease, one of the neurodegenerative diseases, shows the progressive senescence of neural progenitor/stem cells. N-Stearoyl-L-tyrosine (NsTyr) showed neuroprotective effect against chronic brain ischemia in previous reports. In the present study, we find the antisenescent effects of NsTyr-2K in NSPCs induced by A*β*_1–42_ *in vitro*. Cell viability of NSPCs was evaluated by CCK8 assay; SA-*β*-gal staining was used to evaluate senescence of NSPCs. CB receptors were detected by immunohistochemistry in NSPCs. AM251 or AM630 was used to offset the anti-senescence effects afforded by NsTyr-2K. The positive rate of SA-*β*-gal staining was significantly increased in NSPCs after incubation with A*β*_1–42_ for 9 days. CB receptors were found on the surface of NSPCs. The expression level of CB1 receptors was significantly decreased in NSPCs after incubation with A*β*_1–42_. This phenomenon was reversed dose-dependently by NsTyr-2K. NsTyr-2K attenuated A*β*_1–42_ induced NSPCs senescence dose-dependently, and its antisenescence effect was completely abolished by AM630. A*β*_1–42_ dose-dependently increased the prosenescence molecules p16 and Rb. Their expression was inhibited by NsTyr-2K dose-dependently and blocked by AM630 in NSPCs. These results suggest that NsTyr-2K can alleviate the senescence of NSPCs induced by A*β*_1–42_ via CB2 receptor.

## 1. Introduction

Alzheimer's disease (AD), a neurodegenerative disease, is characterized with neurofibrillary tangles, the progressive loss of neuron, and insoluble inclusion of amyloid plaques [1]. Accumulation of the amyloid *β*-peptide is believed to be the initial trigger for neurogeneration. A*β*_1–42_ peptide is more prone to assemble to oligomer and fibril structure than A*β*_1–40_. Small soluble amyloid oligomers deriving from a variety of proteins in very different tissues have recently been suspected to lead to pathologies in many amyloid diseases, such as AD (A*β*), Parkinson's (*α*-synuclein), Huntington's (huntingtin), type-2 diabetes (islet amyloid polypeptide, IAPP), and prion diseases [2]. Although considerable energy and money have been spent trying to find new targets or medicines for the treatment of AD, there are still no drug treatments available that can provide a cure for Alzheimer's disease [3]. Theoretically, damaged or dead neural cells could be replaced by transplanted cells. Therefore, stem cell research will be a major driver in the search for an AD cellular therapy. Endogenous neural stem/progenitor cells (NSPCs) within adult subventricular zone (SVZ) or subgranular zone (SGZ) remain the ability to self-renew and have multilineage potential, differentiating into new neurons or glia. The process of neurogenesis is robust in young and decreases exponentially in aged rodents or primates [4]. Neural stem/precursor cells can be intravenously administered and then migrate into brain damaged areas and induce functional recovery [5].

Neurogenesis, highly sensitive to a hostile microenvironment as the result of stress and aging, is critical for the maintenance of normal learning and memory [6]. It has been reported that there existed increasing number of immature neuronal cells in the brains of patients with AD, compared with the brains of age-matched control subjects [7]. Our previous reports suggested that A*β*_1–42_ oligomer accelerates the senescence of NPSCs via formylpeptide receptor 2 after long-term incubation [8].

Senescent cells, no longer response to external stimuli, remain viable and metabolically active for a long period of time despite their inability to multiply [8]. Therefore, to avoid the senescence of NSPCs in neurodegenerative patients may help the maintenance of neurogeneration in their brain. Endocannabinoid system (ECS) has been shown to be implicated in different pathophysiological functions including cognition and neurodegeneration [9]. Cannabinoid receptors (CB) are widely distributed in the central nerve system. The agonists of CB1 receptor and (or) CB2 receptors both have potential neuroprotective effects on neurodegenerative diseases and brain stroke [10].

The endogenous arachidonate-based lipids, anandamide (N-arachidonoylethanolamide, AEA) and 2-arachidonoylglycerol (2-AG), are known as “endocannabinoids.” Endocannabinoids are taken up by a transporter on the glial cell and primary degraded by fatty acid amide hydrolase (FAAH) or monoacylglycerol lipase (MAGL) [11]. N-Fatty-acyl amino acids exist as endogenous substances and may have a role in regulating tissue functions as endocannabinoid. Among the AEA analogues, N-fatty-acyl amino acids with a long-chain fatty acyl structure have emerged as the most attractive candidates. It has been reported that N-stearoyltyrosine (NsTyr, one of synthesized analogues of AEA) protected primary cortical neurons against A*β*_1–40_ induced injury through inhibiting endocannabinoid degradation via FAAH [12].

Accumulation of misfolded protein aggregates may lead to the brain organ failure during the process of neurodegeneration. In this study, we showed that A*β*_1–42_ oligomer promoted the senescence of cultured NSPCs at 1 *μ*M after long-term incubation. We also provided evidence suggesting that NsTyr-2K inhibits NSPCs senescence via CB2 receptor. This finding may provide us new insights into the potential therapy of stem cell for neurodegeneration including AD.

## 2. Material and Methods

### 2.1. Experimental Animals

Pregnant Sprague-Dawley (SD) rats (350 ± 10 g, grade II, certification number SCXK 2007-0005) were purchased from the Shanghai SLAC Laboratory Co. Ltd. The experimental protocol used in this study was approved by the Ethics Committee for Animal Experimentation and was conducted in strict accordance with the Guidelines for Animal Experimentation of Shanghai Jiao Tong University (Shanghai, China).

### 2.2. Reagents and Drugs

Dulbecco's modified Eagle's medium (DMEM)/F12, B27 Supplement, basic fibroblast growth factor (bFGF), and epidermal growth factor (EGF) were purchased from Invitrogen Corporation (Carlsbad, CA, USA). Cell counting kit-8 (CCK-8) assay was purchased from Beyotime Institute of Biotechnology (China). A*β*_42_ peptide was purchased from AnaSpec incorporated company. NsTyr-2K was kindly provided by Professor Lu Yang (Shanghai JiaoTong University, Shanghai, China). The polyclonal antibodies against Rb, p-16, and GAPDH were purchased from Proteintech (Campbell Park, Chicago, USA). The antibodies against CB1 and CB2 receptor were purchased from Abcam Inc. (Abcam, Cambridge, UK) and Santa Cruz Biotechnology (Santa Cruz, CA, USA), respectively. Senescence *β*-Galactosidase Staining Kit was purchased from Beyotime Institute of Biotechnology (Shanghai, China). All other reagents used were of analytical grade. NsTyr-2K was diluted in cell culture medium before use.

### 2.3. Cell Culture

Neural stem/progenitor cells (NSPCs) were grown from dissociated hippocampal tissue of 14 d Sprague-Dawley rat embryos in Dulbecco's modified Eagle's medium (DMEM)/F12 containing B27 Supplement with 20 ng/mL bFGF, 20 ng/mL EGF. The NSPCs were kept in an incubator at 37°C in a 5% CO_2_ fully humidified atmosphere and were fed every 3-4 days with half-fresh medium. The NSPCs grew into floating neurospheres after 7 days* in vitro*. The neurospheres were dissociated into single cell and passaged every 5 days. For adherent monolayer cultures, methods have been previously described in detail [13]. Cells were plated on dishes percolated with poly-D-lysine hydrobromide (PDL) and laminin containing above-mentioned medium.

### 2.4. Immunohistochemistry

Cells grown on PDL/laminin-coated coverslips were fixed with 4% paraformaldehyde for 30 min at room temperature (RT) and rinsed with PBS three times before being permeabilized in 1% Triton X-100 for 10 min. Nonspecific antibody binding sites were blocked by incubating with normal goat serum for 2 h at RT before labeling with primary antibodies overnight at 4°C. Primary antibodies included the rabbit anti-CB1 antibody (AbCam Limited, Cambridge, UK, 1 : 200) or rabbit anti-CB2 antibody (Cell Signaling Technology, 1 : 200). After washing three times in PBS for 5 min, sections were reacted for 1 h at 37°C in the dark with second antibodies. The second antibodies included AlexaFluor 488 goat anti-rabbit IgG and DyLight 694 goat anti-rabbit IgG (1 : 500). Nuclei were stained with 40, 6-diamidino-2-phenylindole (DAPI). After final washing, the stained slides were mounted with Olympus DP control and DP software.

### 2.5. Oligomeric A*β*_1–42_ Preparation and Neurosphere Proliferation Assay

Oligomeric A*β*_42_ peptide is prepared as described previously. A*β*_1–42_ peptide was first dissolved to 1 mM in 100% hexafluoroisopropanol (HFIP), HFIP was removed under vacuum, and the peptide was resuspended in dimethyl sulfoxide (DMSO) to 5 mM. Dulbecco's modified media (DMEM) were added to bring the peptide to a final concentration of 100 *μ*M and incubated at 4°C for 24 h. The solution was centrifuged at 12,000 g for 20 min, and the supernatant was stored at −20°C before use. After trypsinizing neurospheres, NSCs were resuspended in proliferation medium. The cells were seeded into 96-well plates at a density of 104 cells per well. The drugs were added when the plates were seeded. After incubation for 6 or 9 days, cell proliferation in 96-well plates was measured by a commercially Cell Counting Kit (CCK-8, Beyotime Institute of Biotechnology, China). 10 *μ*L CCK-8 reagent was added to each well and incubated at 37°C for 4 h. Absorbance was measured at 450 nm by using a spectrophotometer. Each experiment was performed in triplicate and repeated at least three times.

### 2.6. SA-*β*-Gal Assay

Senescence was determined by the detection of senescence-associated *β*-galactosidase (SA-*β*-gal) activity using a *β*-Galactosidase Staining Kit (Beyotime Institute of Biotechnology, China). Briefly, cells were plated on PDL-coated coverslips. After treatment with 1 *μ*M A*β*_1–42_ for 9 days, NSPCs were washed three times with PBS and fixed for 30 min at room temperature with fixative solution. After incubation with the staining solution overnight at 37°C, the absolute numbers of SA-*β*-gal+ cells were quantified by counting six random fields per slides. AM251 or AM630 was incubated, respectively, with NSPCs 1 h prior to and during A*β*_1–42_ oligomers insult. NSPCs were treated with different inhibitors in the presence or absence of NsTyr-2K to determine which CB receptor antagonists could block the antisenescence provided by 1 *μ*M NsTyr-2K.

### 2.7. Western Blot

After incubation with NsTyr-2K for 24 h, NSPCs were lysed with RIPA lysis buffer. Total proteins from cell lysates were denatured at 100°C for 5 min and 20 *μ*g of protein was electrophoresed on a 10% SDS-polyacrylamide gel and transferred to a Polyvinylidene Fluoride (PVDF) membrane. The membranes were blocked at room temperature for 1 h in PBS containing 0.5% Tween 20 and 5% BSA and then incubated for 2 h at 37°C with antibody diluted in PBS containing 0.05% Tween 20. After washing, the membranes were incubated for 1 h at 37°C with the appropriate horseradish peroxidase-labeled secondary antibody (Proteintech, Chicago IL) diluted in PBS containing 0.05% Tween 20 and the proteins visualized by enhanced chemiluminescence detection (Pierce, Rockford, IL, USA). GAPDH was used as the internal control.

### 2.8. Statistical Analysis

Data collected from 3~6 independent experiments were used to calculate means, which are expressed as mean ± SD. SPSS statistical software 16.0 for Windows was used and statistical significance was evaluated by using one-way ANOVA and the SNK test. Statistical significance was assumed if *P* < 0.05.

## 3. Results

### 3.1. Oligomeric A*β*_1–42_ Detected by SDS-PAGE

**Figure 1:**
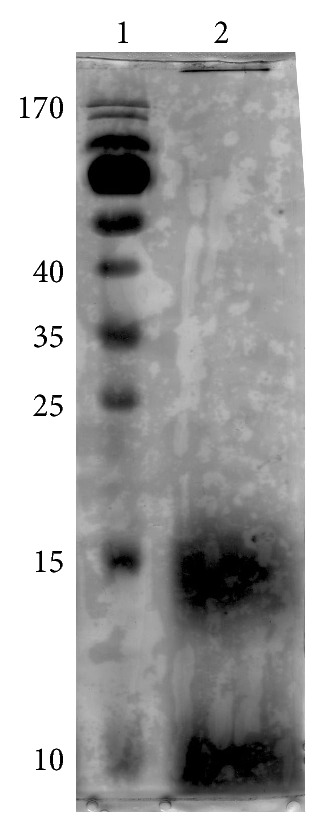
Assembled A*β*_1–42_ oligomer detected by SDS-PAGE. (1) Marker, (2) oligomeric A*β*_1–42_. Analyzed by SDS-PAGE followed by silver staining.

Assembled oligomeric A*β*_1–42_ peptide was detected by SDS-PAGE. As shown in Figure 1, the first lane is marker, and the second lane is oligomeric A*β*_1–42_. We can find that the most of oligomer was found at 16–25 kD (Figure 1).

### 3.2. CB Receptor Detected on the Surface of NSPCs

**Figure 2:**
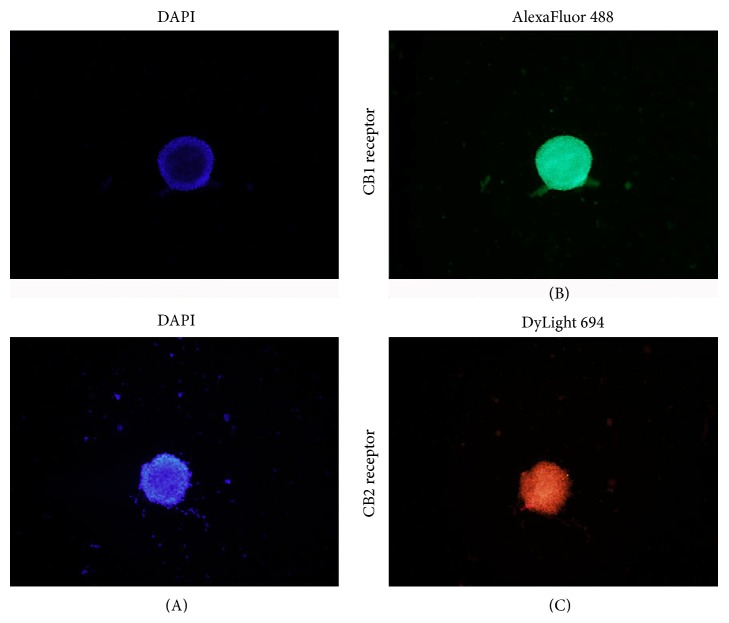
Detection of CB1 and CB2 receptor immunoreactivity in neurosphere. (A) Neurosphere was counterstained with DAPI (blue). (B) Neurosphere was immunolabeled with anti-CB1 antibodies and AlexaFluor 488 goat anti-rabbit immunoglobulins (green). (C) Neurosphere was immunolabeled with anti-CB2 antibodies and DyLight 694 goat anti-rabbit IgG (red) original magnification, 100x.

CB1 and CB2 receptors were detected in human brain, spinal cord. In the present study, we demonstrated that both CB1 and CB2 receptors are expressed in neurosphere consisting of NSPCs. CB1 receptors were stained with AlexaFluor 488 (green) and CB2 receptors were stained with DyLight 694, respectively. Their nuclei were both counterstained with DAPI (blue) (Figure 2).

### 3.3. Effects of A*β*_1–42_ on the Viability of NSPCs after Long-Term Incubation

**Figure 3:**
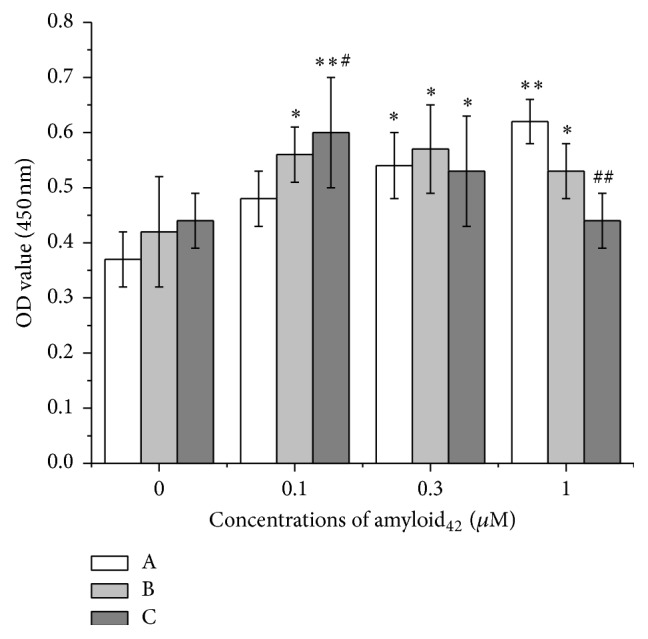
The time course of NSPCs viability after coincubation with different concentrations of A*β*_1–42_. Cell viability was measured by the CCK-8 assay. The concentration of A*β*_1–42_ was 0.1, 0.3, and 1 *μ*M, respectively. The error bars represent the standard error of the mean (S.E.M.) of at least three experiments (*n* = 3). ^*∗*^ *P* < 0.05, ^*∗∗*^ *P* < 0.01 versus control group. ^#^ *P* < 0.05, ^##^ *P* < 0.01 versus 0.1 *μ*M A*β*_1–42_ group. (A) Six days, (B) 9 days, and (C) 12 days.

To analyze the effect of A*β*_1–42_ on proliferation, NSPCs were treated with A*β*_1–42_ (0.1, 0.3, or 1 *μ*M) for different time. The viability of cells was measured at 6, 9, and 12 days using the CCK-8 assay. A*β*_1–42_ dose-dependently promoted the proliferation of NSPCs after 6 days of incubation. A*β*_1–42_ did not significantly change cell viability of NSPCs after 9 days of incubation. Treatment of NSPCs with A*β*_1–42_ for 12 days significantly inhibited viability of NSPCs in a concentration-dependent manner. Based on our screening results, we chose 1 *μ*M of A*β*_1–42_ to incubate NSPCs for 9 days for the following experiments (Figure 3).

### 3.4. Effect of NsTyr-2K on the Expression Level of CB Receptors during A*β*_1–42_ Insult

**Figure 4:**
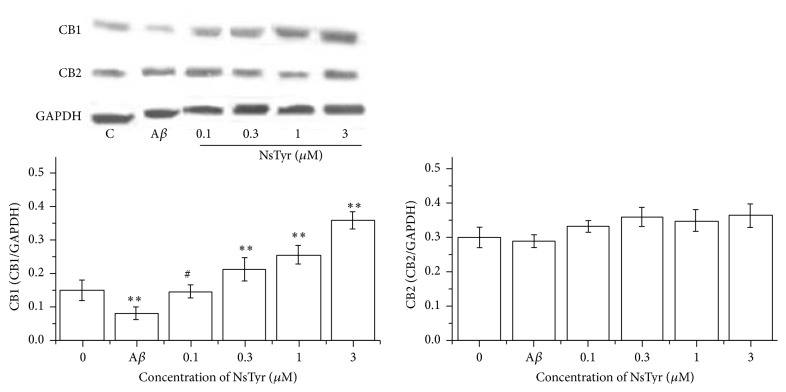
The expression of CB1 and CB2 receptors in NSPCs. The NSPCs were treated with (1) none, (2) 1 *μ*M A*β*_1–42_, (3) 1 *μ*M A*β*_1–42_ + 0.1 *μ*M NsTyr-2K, (4) 1 *μ*M A*β*_1–42_ + 0.3 *μ*M NsTyr-2K, (5) 1 *μ*M A*β*_1–42_ + 1 *μ*M NsTyr-2K, and (6) 1 *μ*M A*β*_1–42_ + 3 *μ*M NsTyr-2K. The expression of CB1 and CB2 was detected by WB. ^*∗∗*^ *P* < 0.05 control group, ^#^ *P* < 0.05 versus A*β*_1–42_ group.

To analyze the effect of NsTyr-2K on the expression level of CB1 and CB2 receptors in NSPCs during A*β* insult, we evaluated their expression by using Western blot. A*β*_1–42_ significantly inhibited the expression of CB1 receptors in NSPCs, and NsTyr-2K dose-dependently reversed the decreasing expression of CB1 receptors induced by A*β*_1–42_. However, A*β*_1–42_ did not have significant effect on the expression levels of CB2 receptors after coincubation. The expression of CB2 receptors did not have been changed by NsTyr-2K in NSPCs during A*β*_1–42_ insult (Figure 4).

**Figure 5:**
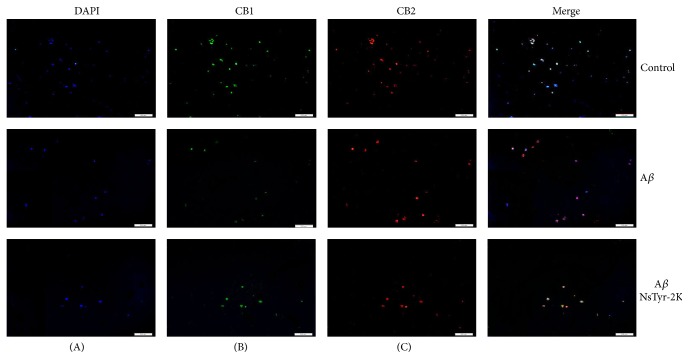
Detection of CB1 and CB2 receptor immunoreactivity in NSPCs. (A) NSPCs were counterstained with DAPI (blue). (B) NSPCs were immunolabeled with anti-CB1 antibodies and AlexaFluor 488 goat anti-rabbit immunoglobulins (green). (C) NSPCs were immunolabeled with anti-CB2 antibodies and DyLight 694 goat anti-rabbit IgG (red) original magnification. (1) None, (2) 1 *μ*M A*β*_1–42_, and (3) 1 *μ*M A*β*_1–42_ + 1 *μ*M NsTyr-2K, 100x.

Immunofluorescence assays confirmed the same results. CB1 receptors were stained with AlexaFluor 488 (green) and CB2 receptors were stained with DyLight 694, respectively. Their nuclei were both counterstained with DAPI (blue). The expression level of CB1 receptors had a significant decrease after A*β*_1–42_ insult, and CB2 receptors did not have been obviously changed (Figure 5).

### 3.5. Effects of NsTyr-2K on the Viability of NSPCs after Long-Term Incubation

**Figure 6:**
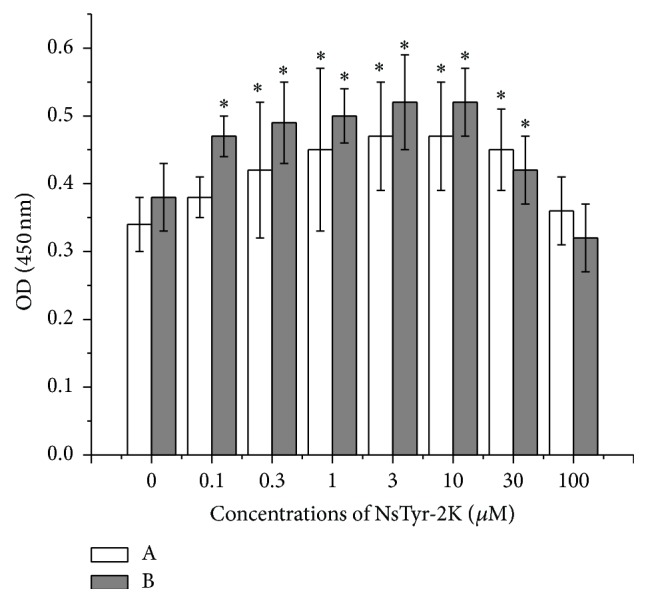
The time course of NSPCs viability after coincubation with different concentrations of NsTyr-2K. Cell viability was measured by the CCK-8 assay. The concentration of NsTyr-2K was 0.1, 0.3, 1, 3, 10, 30, and 100 *μ*M, respectively. The error bars represent the standard error of the mean (S.E.M.) of at least three experiments (*n* = 3). ^*∗*^ *P* < 0.05, ^*∗*^ *P* < 0.05 versus control group. (A) Six days, (B) 9 days.

NsTyr-2K dose-dependently improved NSPCs viability after incubation for 6 days. The maximum proliferative capacity was observed in NSPCs after incubation with 3~10 *μ*M NsTyr-2K for 9 days (Figure 6).

**Figure 7:**
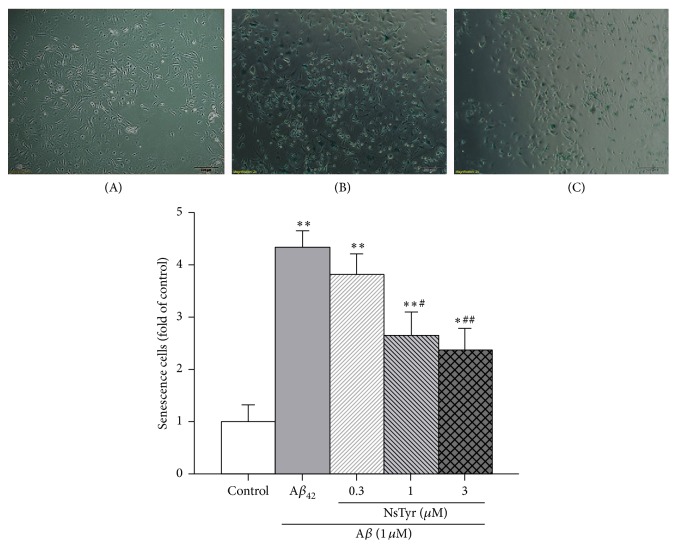
The effects of NsTyr-2K on the numbers of SA-*β*-gal positive cells of NSPCs after A*β*_1–42_ insult for 9 days: (A) control, (B) A*β* group, and (C) A*β*_1–42_ insult + NsTyr-2K. Quantification by densitometric scanning was presented below. The error bars represent the standard error of the mean (S.E.M.) of at least three experiments (*n* = 3). ^*∗*^ *P* < 0.05, ^*∗∗*^ *P* < 0.01 versus control group. ^#^ *P* < 0.05, ^##^ *P* < 0.01 versus A*β*_1–42_ group.

After NSPCs were subjected to 1 *μ*M A*β*_1–42_ for 9 days, the number of positive SA-*β*-gal stainings in NSPCs was increased approximately 4.2-fold. The cells of control group were transparent and plump, but the cells of A*β*_1–42_ group showed the flattened and enlarged morphology. The 0.3–3 *μ*M NsTyr-2K significantly protected NSPCs against cellular senescence induced by A*β*_1–42_ (Figure 7).

### 3.6. Influence of Selective Receptor Antagonist on the Antisenescence Effects of NsTyr-2K

**Figure 8:**
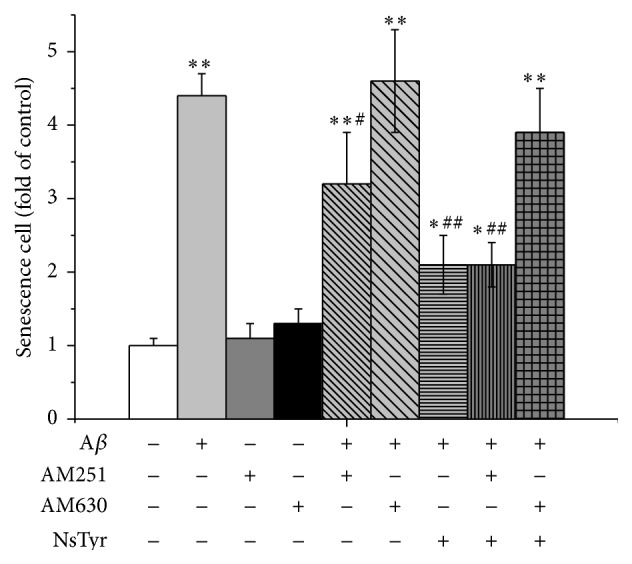
The effects of selective receptor antagonist on the number of senescence cells. The NSPCs were treated with (1) none, (2) A*β*_1–42_, (3) AM251, (4) AM630, (5) A*β*_1–42_ + AM251, (6) A*β*_1–42_ + AM630, (7) A*β*_1–42_ + NsTyr-2K, (8) A*β*_1–42_ + AM251 + NsTyr-2K, and (9) A*β*_1–42_ + AM630 + NsTyr-2K. ^*∗*^ *P* < 0.05, ^*∗∗*^ *P* < 0.01 versus control group. ^#^ *P* < 0.05, ^##^ *P* < 0.01 versus A*β*_1–42_ group.

Since activation of CB1 receptor or CB2 receptor contributes to the neuroprotective effect against focal cerebral ischemia in rats, we tested whether CB1 or CB2 receptor antagonist worsens the antisenescence of Nstry-2K in NSPCs during A*β*_1–42_ insult. 1 *μ*M AM630 or AM251 produced no significant effect during A*β*_1–42_ insult (*P* > 0.05). Those results suggest that cannabinoid receptor antagonists did not directly exacerbate NSPCs senescence during A*β*_1–42_ insult. There was not statistically significant difference between the NsTyr-2K group and the coincubation of NsTyr-2K and AM251 during A*β*_1–42_ exposure (*P* > 0.05). 1 *μ*M AM630 (a potent and specific inhibitor of CB2 receptor) did not protect NSPCs against A*β*_1–42_ insult (*P* > 0.05). However, AM630 statistically ablated the antisenescent effect offered by NsTyr-2K during A*β*_1–42_ insult (Figure 8).

### 3.7. Effect of NsTyr-2K on the Expressions of Prosenescence Molecules

**Figure 9:**
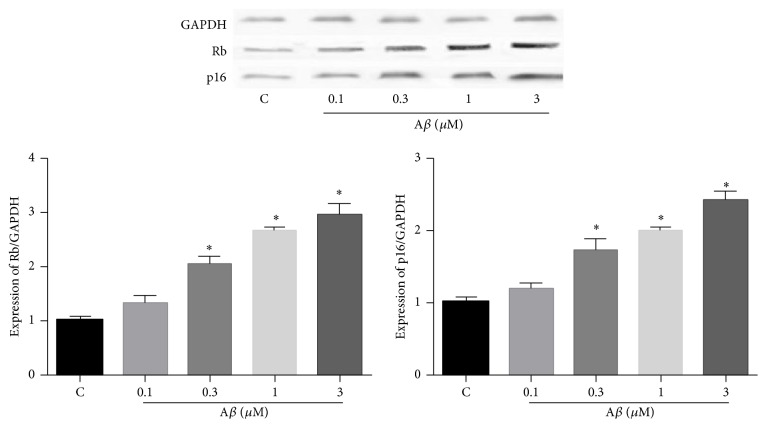
The influence of A*β*_1–42_ on the expression of Rb1 and p16 in NSPCs. The NSPCs were treated with (1) none, (2) 0.1 *μ*M A*β*_1–42_, (3) 0.3 *μ*M A*β*_1–42_, (4) 1 *μ*M A*β*_1–42_, and (5) 3 *μ*M A*β*_1–42_. The expression of Rb1 and p16 was detected by WB. ^*∗*^ *P* < 0.05 versus control group.

To further confirm the effect of NsTyr-2K, we also monitored the expressions of prosenescence molecules p16 and Rb. Rb and p16 can indicate the senescence of cells. A*β*_1–42_ induced the expression of Rb and p16 dose-dependently after 9 days of incubation in NSPCs; these results further confirmed that A*β*_1–42_ induced the senescence of NSPCs after long-term incubation (Figure 9).

**Figure 10:**
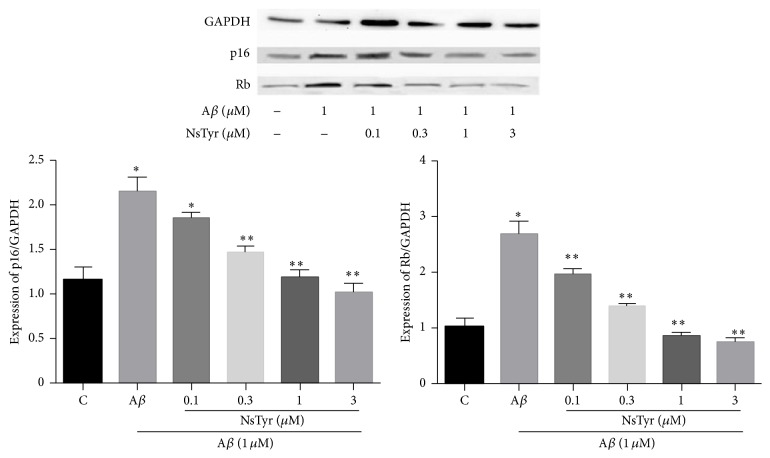
The influence of NsTyr-2K on the expression of Rb1 and p16 in NSPCs which coincubated with A*β*_1–42_. The NSPCs were treated with (1) none, (2) 1 *μ*M A*β*_1–42_, (3) 1 *μ*M A*β*_1–42_ + 0.1 *μ*M NsTyr-2K, (4) 1 *μ*M A*β*_1–42_ + 0.3 AM251, (5) 1 *μ*M A*β*_1–42_ + 1 *μ*M NsTyr-2K, and (6) 1 *μ*M A*β*_1–42_ + 3 *μ*M NsTyr-2K. The expression of Rb1 and p16 was detected by WB. ^*∗*^ *P* < 0.05 versus control group, ^*∗∗*^ *P* < 0.05 versus A*β*_1–42_ group.

**Figure 11:**
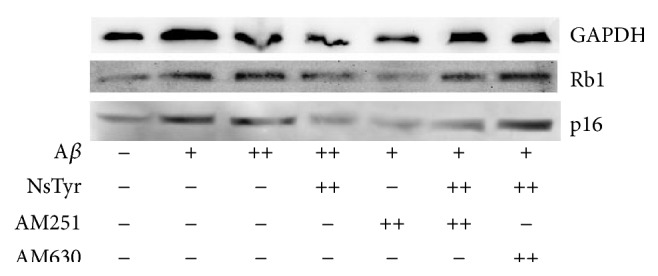
The influence of selective receptor antagonist on the expression of Rb1 and p16 in NSPCs. The NSPCs were treated with (1) none, (2) 0.3 *μ*M A*β*_1–42_, (3) 1 *μ*M A*β*_1–42_, (4) A*β*_1–42_ + NsTyr-2K, (5) A*β*_1–42_ + AM251, (6) A*β*_1–42_ + NsTyr-2K + AM251, and (7) A*β*_1–42_ + NsTyr-2K + AM630. The expression of Rb1 and p16 was detected by WB.

NsTyr-2K dose-dependently inhibited the expression of Rb and p16 induced by A*β*_1–42_ which suggested that NsTyr-2K effectively alleviated the cellular senescence of NSPCs (Figure 10). Additionally, AM630 significantly ablated the antisenescent effect in NSPCs offered by NsTyr-2K during A*β*_1–42_ insult (Figure 11). Therefore, these results further indicated that NsTyr-2K inhibits the senescence of NsTyr-2K by CB2 receptor.

## 4. Discussion

AD is a neurodegenerative disease characterized by progressive loss of neuron, neurofibrillary tangles, and insoluble inclusion of amyloid plaques [1]. Stimulation of endogenous neurogenesis or transplantation of NSPCs has been considered in the therapy of cognitive impairments [14]. Since disturbed brain microenvironment hampers the neurogenesis of AD patients, exogenesis NSPCs transplantation has limited therapeutic benefits [15].

To explore the effect of A*β* on the cell viability of NSPCs, we incubated NSPCs with A*β*_1–42_ and measured the cell viability at 6, 9, and 12 days. We chose 1 *μ*M of A*β*_1–42_ as the highest concentration to evaluate the cell viability in this experiment which was much lower compared with other researches. A*β*_1–42_ dose-dependently improved the viability of NSPCs dose-dependently after incubation for 6 days. After 9 days of incubation, A*β*_1–42_ did not significantly inhibit viability in a concentration-dependent manner. However, A*β*_1–42_ inhibited the proliferation of NSPCs in a dose-dependent manner after 12 days of incubation. These results suggest that A*β*_1–42_ promotes the proliferation of NSPCs at the early stage and inhibits cell viability after long-term incubation. Additionally, A*β*_1–42_ inhibited the expression of CB1 receptors but had no effect on the expression of CB2 receptor significantly. CB2 receptor seems to be a more suitable target for the treatment of neural damage induced by A*β*. A selective agonist of receptor usually downregulates the corresponding receptor to avoid overstimulation via receptor internalization. Our results suggest that A*β*_1–42_ does not directly bind with CB2 receptor in NSPCs. Whether A*β*_1–42_ binds with CB1 receptor directly or indirectly is still under our exploration.

CB1 is one of the most abundant G-protein-coupled receptors in the central nervous system. It has been reported that endocannabinoids act as neurogenic niche cues during cortical development via CB1 receptor. In addition, CB1 receptor is involved in the control of neural cell proliferation/survival decision. CB2 receptor was found in the undifferentiated NPSCs in the brain, which has been proved that CB2 receptor promotes neural progenitor cell proliferation via the activation of the PI3K/Akt/mTORC1 axis. Therefore, cannabinoid receptors play important roles in progenitor/stem cell proliferation and differentiation.

NsTyr-2K, an anandamide (AEA) analogue, is similar to not only AEA structurally but also biological activity. AEA has been demonstrated to have direct neuroprotective effects on neurons against A*β* toxicity [16]. Therefore, we hypothesized that NsTyr-2K might exert antisenescent effect against NSPCs senescence induced by amyloid. Oligomeric A*β*_1–42_ significantly promoted the number of positive numbers of SA-*β*-gal stainings in NSPCs after 9 days of incubation. After they were subjected to NsTyr-2K, the senescent numbers of NSPCs were significantly reduced. Taken together, these results suggest that NsTyr-2K can inhibit the senescent effect induced by A*β*_1–42_ in NSPCs.

Activation of CB1 receptor plays an important role in rapid ischemic tolerance, and activation of CB2 receptor contributes to the delayed neuroprotective effect against focal cerebral ischemia in rats. We tested that the antisenescent effect of NsTyr-2K against A*β*_1–42_ whether via CB1 or CB2 receptor. We found that 1 *μ*M AM630 significantly inhibited the antisenescent effect of NsTyr-2K, but 1 *μ*M AM251 had no significant effect. These results suggest that CB2 receptor may play the key role in the antisenescent effects of NsTry-2K in NSPCs.

Retinoblastoma protein (Rb) and protein 16 (p16) are important signaling proteins of replicative senescence. p16 is a cyclin-dependent kinase inhibitor that acts upstream of Rb to arrest cell cycle. The amount of their expression indicates the serious degree of senescence cells. Rb and p16 both were significantly increased after incubation with 1 *μ*M of A*β*_1–42_ after 9 days of incubation. These results further confirmed that A*β*_1–42_ can induce cellular senescence of NSPCs after long-term incubation. NsTyr-2K significantly inhibited the expression of Rb and p16 induced by A*β*_1–42_. These results further suggest that NsTyr-2K has protective effect against the senescence induced by A*β*_1–42_ in NSPCs. In addition, AM630 offset the antisenescent effect of NsTyr-2K by improving the expression of Rb and p16 in NSPCs. AM251 failed to reverse the antisenescent effect of NsTyr-2K.

In summary, present study demonstrates that NsTyr-2K can effectively protect NSPCs against senescence induced by A*β*_1–42_ within a given range of concentrations. Its antisenescence effect is possibly mainly mediated by the activation of CB2 receptor in NSPCs.
